# The effect of the Covid‐19 pandemic on illness perceptions of psoriasis and the role of depression: Findings from a cross‐sectional study

**DOI:** 10.1002/ski2.145

**Published:** 2022-07-03

**Authors:** Georgia Lada, Hector Chinoy, Peter S. Talbot, Richard B. Warren, C. Elise Kleyn

**Affiliations:** ^1^ Dermatology Centre Salford Royal NHS Foundation Trust Manchester National Institute for Health Research Biomedical Research Centre The University of Manchester Manchester UK; ^2^ Division of Neuroscience and Experimental Psychology Faculty of Biology, Medicine and Health The University of Manchester Manchester UK; ^3^ National Institute for Health Research Manchester Biomedical Research Centre Manchester University NHS Foundation Trust The University of Manchester Manchester UK

## Abstract

**Background:**

Illness perceptions in psoriasis have an impact on adherence and disability. Changes in dermatological healthcare provision during the Covid‐19 pandemic and distress may have affected illness perceptions in psoriasis patients.

**Objectives:**

To test whether illness perceptions about psoriasis changed during the first year of the Covid‐19 pandemic compared to pre‐pandemic in a tertiary population with psoriasis and whether pandemic effects differed depending on depressive burden, given this population's high depression prevalence.

**Methods:**

In a cross‐sectional survey of *n* = 188 tertiary patients with dermatologist‐confirmed psoriasis recruited before and during the pandemic, eight illness perceptions domains were assessed using the Brief‐Illness Perceptions Questionnaire (BIPQ). Presence of depression was assessed with the Hospital Anxiety and Depression Scale (HADS).

**Results:**

Beliefs about treatment control and patients' understanding of psoriasis were significantly worse in patients responding during the pandemic compared to before Covid‐19. These differences were greater when depression was absent (treatment control: adjusted *p* < 0.001; coherence: adjusted *p* = 0.01). However, participants during the pandemic felt less emotionally affected (adjusted *p* = 0.02) and concerned (adjusted *p* = 0.007) about psoriasis, independently of depression.

**Conclusions:**

We found diverse pandemic effects on illness perception domains in psoriasis. Uncertainty and reduced healthcare access may drive poorer treatment and coherence beliefs during Covid‐19. These beliefs can hinder patients' health‐promoting behaviours and may explain the high pandemic non‐adherence reported previously in psoriasis. Appropriate interventions are needed to establish positive long‐term cognitions and improve psoriasis management, for example, using the PsoWell patient materials. Dermatology services should invest in engaging and educating patients regardless of concurrent psychological distress.

## Graphical abstract

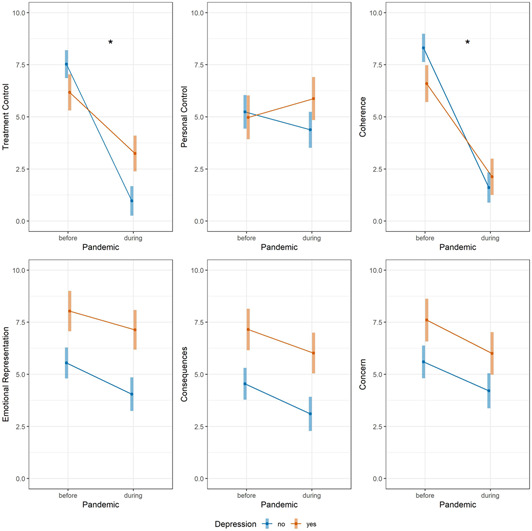

1

**What is already known about this topic?**

- Illness perceptions in psoriasis affect non‐adherence and disability. Some illness perception domains have been associated with depression.
- Non‐adherence in psoriasis during the Covid‐19 pandemic was present in up to 68.5% of patients. Psoriasis patients, in particular in tertiary care, faced multiple challenges during the pandemic, including a reduction in healthcare access, fear of infection, shielding, and distress, which may have had an impact on their perceptions about the disease.

**What does this study add?**

- Treatment control beliefs and understanding of psoriasis were worse among patients in the first year of the Covid‐19 pandemic compared to pre‐pandemic. Worsening of these perceptions was greater in the absence of depression.
- Patients felt less concerned and emotionally affected by psoriasis during the pandemic, regardless of depressive burden.
- Addressing negative illness beliefs with appropriate interventions (e.g., PsoWell patient materials) may help adherence in psoriasis in the post‐pandemic transition period.

## 2 INTRODUCTION

The first year of the Covid‐19 pandemic significantly affected healthcare provision and access. During lockdown in England, total dermatology outpatient appointments halved, despite the introduction of remote consultations.^1^ Patients with moderate‐to‐severe psoriasis, who are often under treatment with conventional systemic and biologic drugs and have pre‐pandemic depression rates of 19%–28%,^2^ may have faced particular challenges in the form of shielding measures and higher perceived infection risk.

Changes to healthcare provision as well as pandemic‐driven feelings of control loss and uncertainty^3^ may have negatively affected core illness beliefs in vulnerable patients, in particular their sense of control over their illness. Furthermore, the non‐adherence rate in psoriasis during the pandemic has been reported to reach 68.5%.^4^, ^5^ As negative illness perceptions are a major cause of non‐adherence and long‐term predictor of disease burden, distress, and future disability in psoriasis, identifying and addressing them early is important.^6^, ^7^, ^8^, ^9^ Aligning with the theoretical and empirical evidence of negative cognitive bias in depression,^10^ negative illness perceptions in some domains have been associated with higher depressive burden.^11^

We hypothesised that patients' perceptions about psoriasis changed during the first year of the Covid‐19 pandemic compared to before the pandemic in a tertiary population. Furthermore, we investigated whether pandemic effects differed depending on patients' depressive burden.

## 3 MATERIALS AND METHODS

### 3.1 Procedure and participants

A cross‐sectional survey of tertiary patients with psoriasis was conducted. Data were collected as part of a pre‐existing survey assessing mental health in patients with psoriasis; this study was initiated before the Covid‐19 pandemic and its recruitment and methods have been previously described in detail.^12^ Briefly, patients were recruited from our tertiary Greater Manchester psoriasis and psoriatic arthritis clinics and were eligible provided they had: age of 18–65 years, a dermatologist‐confirmed diagnosis of chronic plaque psoriasis, capacity and willingness to give informed consent, and registration with a general practitioner (GP). We excluded patients who had significant neurocognitive impairment or insufficient command of the English language. We made every effort to include and assist participants with questionnaire completion and clarifications, where appropriate.^12^

A first group of participants had enroled in this pre‐existing study before the first Covid‐19 lockdown initiated in March 2020 in England (September 2019–February 2020).^12^ Patients completed the Brief‐Illness Perceptions Questionnaire (BIPQ) adapted for psoriasis, which is a validated measure assessing eight perception domains.^13^ The presence of depression was assessed using the Hospital Anxiety and Depression Scale (HADS)^14^; psoriasis‐related quality of life was assessed with the Dermatology Life Quality Index (DLQI).^15^ The patients also completed the Quick Inventory of Depressive Symptomatology Self‐Report (QIDS‐SR)^16^ assessing depression severity.

During the first year of the Covid‐19 pandemic (May 2020–February 2021), we recruited a second group of patients, who completed the same questionnaires. Sociodemographic and clinical data were first self‐reported by patients, and medical records were also screened with patients' consent, in order to confirm medical history information and complete missing data. All patients gave written informed consent before enrolment and data collection. All data were collected following review and favourable opinion by the North West—Greater Manchester West Research Ethics Committee (REC) and Health Research Authority approval (REC reference 19/NW/0351, initial approval in July 2019; Covid‐19 amendment SA02, approved in April 2020).

### 3.2 Statistical analysis

Classic statistical tests were used to compare sociodemographic and clinical characteristics between the two groups (participating during vs. participating prior to the pandemic). We investigated differences in seven illness perception domain scores between groups using analysis of variance (ANOVA). To determine whether the effect of the pandemic on illness perceptions depended on depression at time of completion, an interaction term for time of participation*depression was included. A dichotomous depression variable was used, based on a HADS‐Depression subscale cut‐off of 8, which shows optimal screening properties for identifying depression cases.^17^ We controlled for potential confounders, selected based on existing evidence and the degree of heterogeneity between groups: age; gender; coexistent psoriatic arthritis (PsA); other physical comorbidities; and number of biologics ever tried. The latter was used as a proxy for overall lifetime disease burden; Psoriasis Area and Severity Index (PASI) was not performed during Covid‐19. As perceptions regarding illness timeline were negatively skewed and 84.6% of participants scored the maximum, scores were dichotomised in patients who scored maximum and patients who did not, as described previously^18^ and logistic regression was performed using the same covariates as in the linear models.

We corrected for multiple comparisons using the Benjamini‐Hochberg method to control the false discovery rate (FDR). Post‐hoc tests were performed, where the interaction term was significant (*p* < 0.05).

No data were missing for covariates included in models. Missing data for other variables are reported in Table 1. We report effect sizes as ω^2^ for analysis of variance, which has been shown to estimate effect size more accurately and is less biased than generalised and partial η^2^.^19^, ^20^, ^21^, ^22^

**TABLE 1:** Sample characteristics

Baseline characteristics	Missing data (%)		Group 2	*p*‐value
		Group 1Pre‐Covid (*n* = 99)	During Covid‐19 pandemic (*n* = 89)	
Age (years)	0 (0.0)	42.4 (11.4)	50.1 (10.1)	**<0.001**
Gender (f)	0 (0.0)	46 (46.5%)	34 (38.2%)	0.319
Employment status	0 (0.0)			0.781
Employed/student		69 (69.7%)	66 (74.2%)	
Retired		11 (11.1%)	9 (10.1%)	
Unemployed		19 (19.2%)	14 (15.7%)	
Education	1 (0.5)			0.982
High school diploma (or equivalent) or less		47 (48.0%)	43 (48.3%)	
University degree		29 (29.6%)	27 (30.3%)	
Professional degree		22 (22.4%)	19 (21.3%)	
Psoriasis duration (years)	0 (0.0)	25.0 (12.2)	28.2 (12.9)	0.081
Number of lifetime biologics, median (IQR)	0 (0.0)	1.0 (1.0)	1.0 (1.0)	0.079
Treatment for psoriasis	0 (0.0)			0.604
Biologic		78 (78.8%)	69 (77.5%)	
Conventional systemic		8 (8.1%)	7 (7.9%)	
Fumaric acid ester		2 (2.0%)	5 (5.6%)	
Other		11 (11.1)	8 (9.0%)	
DLQI score, median (IQR)	3 (1.6)	3.5 (8.2)	1.0 (7.0)	**0.015**
BMI	1 (0.5)	31.4 (6.7)	33.2 (9.4)	0.130
Smoking	0 (0.0)	21 (21.2%)	12 (13.5%)	0.230
AUDIT‐C	0 (0.0)	3.0 (5.0)	3.0. (5.0)	0.806
Psoriatic arthritis (PsA)	0 (0.0)	30 (30.3%)	46 (51.7%)	**0.004**
Physical comorbidities other than PsA^a^	0 (0.0)	57 (57.6%)	63 (70.8%)	0.083
Probable depression (HADS‐depression score ≥8)	0 (0.0)	37 (37%)	35 (39%)	0.901
QIDS‐SR score	0 (0.0)	8.35 (6.06)	8.89 (5.14)	0.506
HADS‐anxiety score, median (IQR)	1 (0.5)	7.68 (5.43)	7.21 (4.85)	0.532
Lifetime diagnosis of depression	0 (0.0)	40 (40.4%)	31 (34.8%)	0.525
Lifetime diagnosis of any psychiatric disorder	0 (0.0)	48 (48.5%)	42 (47.2%)	0.975

*Note*: All categorical variables are reported as count (%) and all numerical variables as mean (standard deviation), unless otherwise specified. Descriptive statistics reported in non‐missing data for each cell. *p*‐values < 0.05 are reported in bold.

Abbreviations: AUDIT‐C, alcohol use disorders identification test for consumption; BMI, body mass index; DLQI; dermatology life‐quality index; HADS, hospital anxiety and depression scale; IQR, interquartile range; QIDS‐SR, quick inventory of depressive symptomatology self‐report.

^a^ Includes cardiovascular, inflammatory and chronic infectious, other severe systemic disease and malignancies, neurological and endocrine disorders.

As a sensitivity analysis, we repeated the analysis using total QIDS‐SR (quick inventory of depressive symptomatology‐self report) scores as an alternative, continuous depression outcome. QIDS‐SR measures depressive symptom severity and its items reflect the DSM‐IV (Diagnostic and Statistical Manual of Mental Disorders) criteria for major depressive disorder (MDD).^16^ Participants completed the QIDS‐SR at the same time as the HADS.

Regarding power considerations, we estimated, using G*power, that, for our two‐way analysis of variance design with five covariates and the interaction as main predictor, we would need a total sample of at least *n* = 171 participants to detect differences with effect size of Cohen's *f* ≥ 0.25 with a 90% power.^23^ All other analysis was performed in *R*.^24^

## 4 RESULTS

### 4.1 Participant characteristics

A hundred and 88 participants completed the survey; *n* = 99 patients prior to the pandemic (September 2019–February 2020) and *n* = 89 during the first year of the pandemic (May 2020–February 2021).

We report sociodemographic and clinical characteristics of the two groups in Table 1. The groups did not differ for gender, employment or education status, psoriasis treatment or personal and family psychiatric history; the pandemic group was older. Dermatology Life Quality Index (DLQI) scores were lower in the group responding during the pandemic (*p* = 0.015); depression and anxiety levels did not differ between the groups. A minority of patients in this group (18.0%, *n* = 16) were living alone during Covid‐19.

### 4.2 Illness perceptions

We found that the pandemic had significant and large effects on treatment control and coherence perceptions. There was significant interaction between depression and time of participation for these domains (treatment control: *p* < 0.001; coherence: *p* = 0.01) (Table 2). Patients felt that treatment helped less and reported poorer understanding of their psoriasis during Covid‐19, with differences during versus before the pandemic being greater among the non‐depressed patients (β 95% confidence intervals (CI) for pandemic effects on treatment control: 6.56 (5.61, 7.52) for non‐depressed and 2.93 (1.71, 4.16) for depressed; on coherence: 6.70 (5.72, 7.67) for non‐depressed and 4.47 (3.22, 5.72) for depressed participants; Table 3).

**TABLE 2:** Effects of time of participation (before/during the Covid‐19 pandemic) and depression on illness perception domain scores

	Group scores, mean (SD)		Group			Depression			Interaction		
									Group * depression		
Illness perceptions domain	Before pandemic	During pandemic	*F*	*p*	ω^2^ (95% CI)	*F*	*p*	ω^2^ (95% CI)	*F*	*p*	ω^2^ (95% CI)
Treatment control	7.06 (2.93)	1.76 (2.16)	133.0	**<0.001**	0.39 (0.28, 0.48)	1.4	**0.342**	<0.01 (<0.01, 0.03)	23.5	**<0.001**	0.07 (0.01, 0.15)
Personal control	4.92 (2.99)	4.86 (3.09)	0.002	0.964	<0.01^a^	1.7	0.314	<0.01 (<0.01, 0.4)	3.7	**0.108**	0.01 (<0.01, 0.07)
Consequences	5.67 (3.19)	4.03 (3.41)	7.4	**0.017**	0.03 (<0.01, 0.09)	37.6	**<0.001**	0.15 (0.07, 0.25)	0.1	0.783	<0.01^a^
Coherence	7.60 (2.98)	1.67 (1.98)	176.7	**<0.001**	0.47 (0.37, 0.56)	2.2	0.235	<0.01 (<0.01, 0.04)	8.5	**0.011**	0.02 (<0.01, 0.08)
Concern	6.46 (3.12)	4.68 (3.15)	9.4	**0.007**	0.04 (<0.01, 0.11)	16.7	**<0.001**	0.07 (0.02, 0.16)	0.05	0.843	<0.01^a^
Emotional representation	6.66 (3.14)	4.85 (3.49)	6.7	**0.022**	0.02 (<0.01, 0.08)	40.4	**<0.001**	0.16 (0.07, 0.26)	0.5	0.546	<0.01^a^
Identity	5.71 (3.07)	4.58 (3.17)	2.4	0.231	0.01 (<0.01, 0.05)	28.2	**<0.001**	0.12 (0.05, 0.22)	0.6	0.526	<0.01^a^

*Note*: All *p*‐values are FDR‐adjusted; *p*‐values < 0.05 before adjustment are reported in bold. For the Timeline domain (binary), beta, 95% CI (*p*‐values) were for group: 0.54, −0.59 to 1.75 (0.45); depression: −0.61, −4.26 to 0.97 (0.24) and interaction: 0.95, −0.83 to 2.87 (0.30).

Abbreiations: CI, confidence intervals; sd, standard deviation.

^a^ Very small values: <0.001, 95% CI (<0.001, <0.001).

**TABLE 3:** Estimated marginal means and post‐hoc tests for illness perceptions scores with significant interaction between depression and time of participation

Illness perceptions domain	No depression				Depression			
	Before pandemic^a^	During pandemic^a^	Beta (95% CI)	*p*‐value	Before pandemic^a^	During pandemic^a^	Beta (95% CI)	*p*‐value
Treatment control	7.53 (6.86–8.19)	0.97 (0.26–1.68)	6.56 (5.61, 7.52)	**<0.001**	6.17 (5.30–7.04)	3.24 (2.38–4.10)	2.93 (1.71, 4.16)	**<0.001**
Personal control	5.24 (4.43–6.05)	4.38 (3.52–5.24)	0.86 (−0.29, 2.02)	0.144	4.98 (3.92–6.03)	5.88 (4.84–6.91)	−0.90 (−2.39, 0.59)	0.233
Coherence	8.31 (7.63–8.99)	1.61 (0.88–2.33)	6.70 (5.72, 7.67)	**<0.001**	6.59 (5.71–7.48)	2.12 (1.25–3.00)	4.47 (3.22, 5.72)	**<0.001**

^a^ Values are presented as estimated marginal means (95% confidence intervals [CI]). Depression cases identified using a hospital anxiety and depression (HADS)—depression subscale cut‐off ≥8. *p*‐values < 0.05 are reported in bold.

**FIGURE 1:**
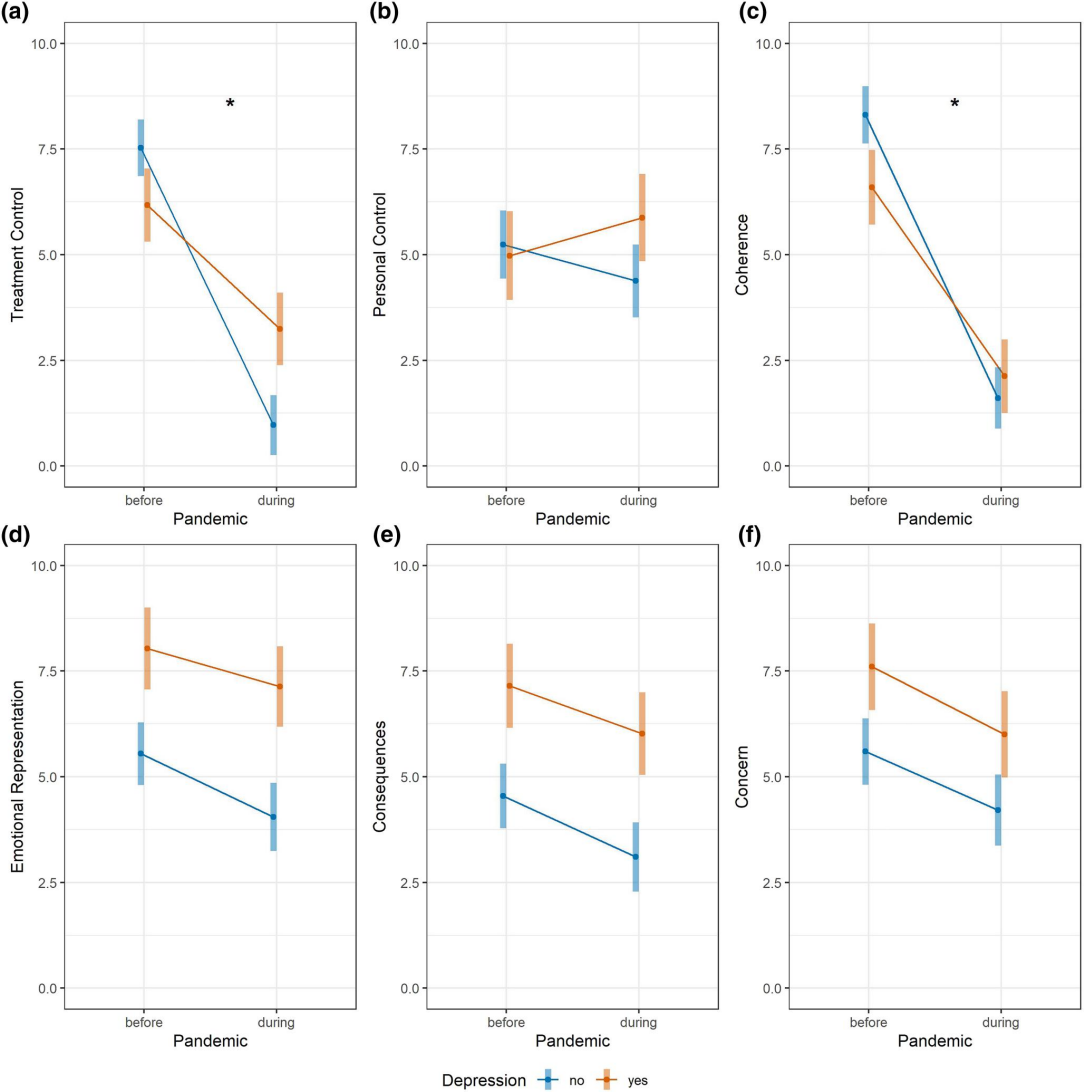
Estimated marginal means and confidence intervals (CIs) for Brief‐Illness Perceptions Questionnaire (BIPQ) scores representing six illness perceptions domains in depressed and non‐depressed patients with psoriasis before and during the Covid‐19 pandemic. Lower BIPQ scores in the three domains of the top row indicate beliefs of poorer (a) treatment control, (b) personal control and (c) understanding of disease. Lower scores in the three domains of the bottom row indicate beliefs of less (d) emotional impact (e) impact on life and (f) concern. Score differences before versus during the pandemic were significantly greater for non‐depressed than depressed patients for treatment control and coherence

Significant associations of smaller effect sizes were observed between time of participation and other illness perception domains. Although non‐depressed individuals generally experienced less personal control over their psoriasis during the pandemic, an inverse trend was observed among depressed patients; this difference in trends between depressed and non‐depressed patients was not statistically significant after FDR control (*ω* ^2^ = 0.01, unadjusted *p* = 0.01; adjusted *p* = 0.11). However, overall patients reported feeling less emotionally affected (emotional representations: *ω* ^2^ = 0.02, *p* = 0.02) and less concerned about their illness (*ω* ^2^ = 0.04, *p* = 0.007) during Covid‐19 (Table 2; Figure 1). Perceptions about illness timeline (chronicity) or identity (how much patients experienced symptoms) did not differ between groups.

When we repeated the analysis using a continuous depression outcome (QIDS‐SR scores), our results did not change in magnitude or significance except for the identity model, where the main effect for the group was borderline significant, however with a similar effect size as for the main analysis (*ω* ^2^ = 0.01, *p* = 0.042) (Supporting Information: Figure S1).

## 5 DISCUSSION

To our knowledge, this is the first report investigating illness perceptions in psoriasis in connection to depression comorbidity during Covid‐19.

We found that patients' beliefs about how well treatment can control their illness as well as their understanding of psoriasis were significantly worse during the pandemic. One possible explanation is reduced access to information and healthcare during the pandemic. Ibrahim and colleagues showed a rapid reduction in dermatology outpatient appointments in England during the first national lockdown, which only partially improved after the end of lockdown.^1^ Although the causes for this were not investigated, both patient infection concerns and a reduction or redistribution of resources within the National Health Service (NHS) may have contributed to this effect.^1^ A German report from the first pandemic year (May–June 2020) suggests less pronounced changes in healthcare provision (about 20% missed appointments), with most cancellations being patient‐initiated.^25^ In a survey during May‐August 2021 in Turkey, inability to visit the hospital was reported as the primary reason for non‐adherence (19.2%) among patients with psoriasis, closely followed by infection‐related anxiety (16.3%) and impaired access to doctors (7.3%) and treatments (7.3%).^5^ However, our results may also reflect wider effects of the pandemic on sense‐making processes and general feelings of uncertainty.^26^

Notably, although depressed patients in our study had poorer illness beliefs pre‐pandemic, the negative pandemic effect over these beliefs was significantly smaller compared to non‐depressed individuals. Evidence from a longitudinal case‐control study in the early months of lockdown showed no change in mental health outcomes among depressed and anxious patients, and even improvement in those with high baseline psychiatric burden.^27^ It is possible that patients with depression have already adapted to more restricted lifestyles and pre‐existing barriers in accessing medical care^28^ or benefited from the slower pace of the pandemic life. Natural regression to the mean may contribute to the observed effects.^27^

Covid‐19 affected other illness perception domains less; we found no significant differences before versus during the pandemic in how much patients were experiencing symptoms from their psoriasis. Depression was associated with the domains of emotional representation, identity and concern, aligning with previous evidence.^11^ These domains, which mainly involve emotional reactions to illness, generally showed improvement in patients during Covid‐19.

It is not clear why emotions about psoriasis as well as dermatology‐related quality of life were generally better in our pandemic group, whereas perceptions about coherence and treatment were significantly worse. It is plausible that patients' concern and emotional attention were predominantly directed towards more imminent, pandemic‐related threats during Covid‐19 rather than chronic, pre‐existing stressors. Furthermore, DLQI largely assesses impairment during interpersonal interactions, activities and performance usually taking place in the social context, and the better scores during Covid‐19‐lockdown are not unexpected.

Our findings support in part and help elucidate prior reports of high non‐adherence in psoriasis during the pandemic.^4^, ^5^ Previous work also found associations between non‐adherence, self‐reported deterioration of psoriasis and poor mental health during Covid‐19.^4^, ^29^

The pandemic effects we detected on treatment beliefs align with these previous results. Negative beliefs about necessity and effectiveness of treatment are major causes of intentional non‐adherence in psoriasis^7^; in contrast, positive beliefs about personal and treatment control over illness predict higher engagement with services.^8^ We found complex relationships between depression and illness perceptions in these domains. During the pandemic, feelings of personal control tended to be better in the presence of depression, contradicting these previous studies. In contrast, depression was significantly associated with experiencing more psoriasis symptoms (identity), which supports the association between self‐reports of worsening psoriasis and depression found in these previous reports. Of note, depression levels were similar between groups (Table 1). This is in partial concordance with a recent review, showing that, during summer 2020, when most of our pandemic participants responded, distress in the general population was near pre‐pandemic levels after an initial increase; although, interestingly, depressive burden may have declined at a slower rate than general distress.^30^

Negative treatment perceptions and deficits in coherence constitute important bottlenecks in patients' self‐management and motivation towards health‐promoting behaviours.^31^ However, there is evidence that these illness perceptions could be modified and changes could be sustainable in the long‐term, by using appropriate interventions which promote patients' health education without causing distress or increasing uncertainty.^31^, ^32^ In psoriasis, the use of PsoWell patient materials is a low‐cost intervention, which has been found to improve illness coherence, treatment and personal control beliefs.^32^ These materials consist of 15 leaflets designed according to health literacy concepts, based on findings of the IMPACT project and the Medical Research Council complex interventions guidance.^33^ They provide signposting for both newly diagnosed and chronically ill patients, deliver key information around the condition, its comorbidities and treatment, give tips for lifestyle management and include adherence‐promoting activities.^32^, ^33^ Techniques improving communication of scientific evidence between healthcare professionals and patients, such as storytelling,^34^ may also be useful.

Our results should be interpreted in the light of some limitations. Our sample consisted of tertiary patients and may not be representative of populations with mild psoriasis course. Second, the cross‐sectional nature of the study does not allow for directionality assumptions regarding the relationship between illness perceptions and depression. Third, we used a between‐group, non‐randomized design, and therefore cannot exclude the influence of residual or unmeasured confounding on the observed associations. In particular, we note the higher age of the pandemic group; as well as the lack of information about psoriasis severity (PASI) at the time of study. Nevertheless, we made efforts to reduce bias in our analysis by controlling for age and other potentially confounding variables which differed between groups, including lifetime biologics use as a proxy for lifetime psoriasis severity. Furthermore, previous reports have found no associations of PASI with patients' beliefs.^6^ Finally, our sample size is relatively small, and the potential for false negative results, in particular for effect sizes Cohen's *f* < 0.25, should be considered when interpreting our findings.

During the current transition period and in future pandemics, it is important for services to invest in engaging and educating patients about psoriasis to achieve better long‐term management of the condition. Since non‐depressed individuals may have experienced greater worsening of their psoriasis perceptions during Covid‐19, it is critical that these strategies target patients regardless of mental health burden.

## AUTHOR CONTRIBUTIONS

**Georgia Lada**: Conceptualisation (lead); Data curation (lead); formal analysis (lead); Investigation (lead); Methodology (lead); Project administration (lead); Resources (supporting); Visualisation (lead); Writing – original draft (lead); Writing – review and editing (equal). **Hector Chinoy**: Conceptualisation (supporting); Funding acquisition (supporting); Investigation (supporting); Methodology (supporting); Project administration (supporting); Resources (supporting); Supervision (equal); Writing – review and editing (equal). **Peter S. Talbot**: Conceptualisation (supporting); Formal analysis (supporting); Investigation (supporting); Methodology (supporting); Project administration (supporting); Resources (Supporting); Supervision (equal); Writing – review and editing (equal). **Richard B. Warren**: Conceptualisation (equal); Funding acquisition (supporting); Investigation (supporting); Methodology (supporting); Project administration (supporting); Resources (supporting); Supervision (equal); Writing – review and editing (equal). **C. Elise Kleyn**: Conceptualisation (equal); Funding acquisition (lead); Investigation (supporting); Methodology (supporting); Project administration (lead); Resources (supporting); Supervision (lead); Writing – review and editing (equal).

## CONFLICT OF INTERESTS

C. Elise Kleyn has received honoraria, consultant and/or research funding from Janssen, Eli Lilly, LEO, Novartis, Abbvie, UCB, Almirall, Pfizer, and L’Oréal. Hector Chinoy has received personal compensation for activities with Novartis, UCB, Lilly, Biogen, Orphazyme as a speaker, advisory board member or consultancy, grants via The University of Manchester from Novartis, UCB and MedImmune, and has received travel support from Abbvie and Janssen. Richard B. Warren has received research grants from AbbVie, Almirall, Amgen, Celgene, Janssen, Lilly, Leo, Medac, Novartis, Pfizer, and UCB and consulting fees from AbbVie, Almirall, Amgen, Arena, Astellas, Avillion, Boehringer Ingelheim, Bristol Myers Squibb, Celgene, DiCE, GSK, Janssen, Lilly, Leo, Medac, Novartis, Pfizer, Sanofi, Sun Pharma, UCB, and UNION. GL has received speaker honoraria from Janssen, Lilly, Leo, and Novartis. Peter S. Talbot has no conflicts of interest.

## ETHICS STATEMENT

All data were collected following review and favourable opinion by the North West ‐ Greater Manchester West Research Ethics Committee (REC) and Health Research Authority approval (REC reference 19/NW/0351, initial approval in July 2019; Covid‐19 amendment SA02, approved in April 2020).

## Supporting information

Supporting Information S1

## Data Availability

Data that support the findings of this study are available upon reasonable request from the corresponding author and are subject to privacy and ethical restrictions.
