# Supplementary material for: The effect of the Covid‐19 pandemic on illness perceptions of psoriasis and the role of depression: Findings from a cross‐sectional study
Source: Skin Health Dis. 2022 Jul 3;2(3):e145. doi: 10.1002/ski2.145 (PMC9435449; doi:10.1002/ski2.145)
Supplement: Supplementary file 1 — Supporting Information S1 [file SKI2-2-e145-s001.docx]

**Supplementary Material**


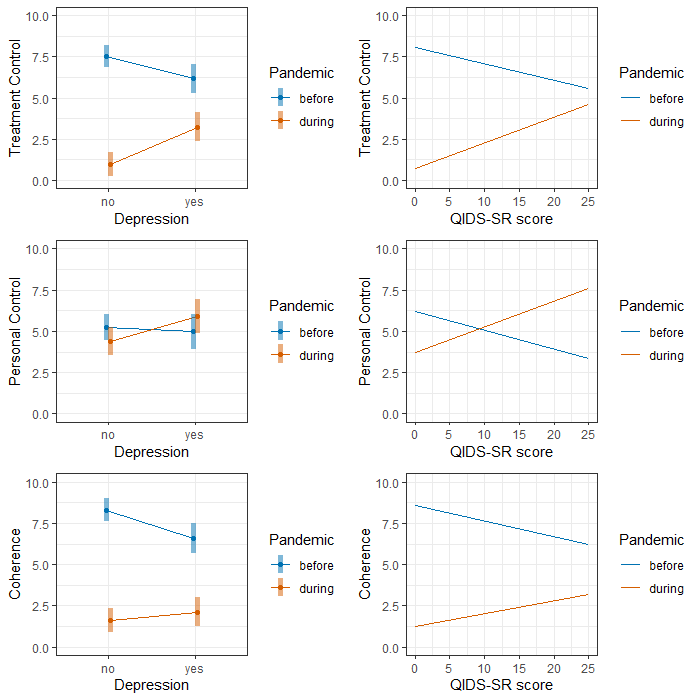


(f)

(e)

(d)

(c)

(b)

(a)

Figure S1: Left (a, b, c): Estimated marginal means and CIs (Confidence Intervals) for BIPQ (Brief-Illness Perceptions Questionnaire) scores representing three illness perceptions domains before and during the Covid-19 pandemic in depressed and non-depressed patients. Depression is assessed as a yes/no outcome, based on a HADS (Hospital Anxiety and Depression Scale)-Depression subscale cut-off ≥8. Right (d, e, f): Trends of mean illness perception scores for different depressive symptom severity scores before and during the pandemic. Depression is assessed as total QIDS-SR (Quick Inventory of Depressive Symptomatology-Self Report) score. Lower BIPQ scores in the three domains from top to bottom indicate beliefs of poorer treatment control, personal control and understanding of disease.
